# The Effects of Transcranial Direct Current Stimulation on Balance and Gait in Stroke Patients: A Systematic Review and Meta-Analysis

**DOI:** 10.3389/fneur.2021.650925

**Published:** 2021-05-25

**Authors:** Ke Dong, Shifeng Meng, Ziqi Guo, Rufang Zhang, Panpan Xu, Erfen Yuan, Tao Lian

**Affiliations:** ^1^Department of Rehabilitation Medicine, First Hospital of Shanxi Medical University, Taiyuan, China; ^2^First Clinical Medical College, Shanxi Medical University, Taiyuan, China

**Keywords:** stroke, balance, transcranial direct current stimulation, meta-analysis, gait

## Abstract

**Objective:** Balance dysfunction after stroke often results in individuals unable to maintain normal posture, limits the recovery of gait and functional independence. We explore the short-term effects of transcranial direct current stimulation (tDCS) on improving balance function and gait in stroke patients.

**Methods:** We systematically searched on PubMed, Web of Science, EMBASE, Cochrane Central Register of Controlled Trials, and Google Scholar for studies that explored the effects of tDCS on balance after stroke until August 2020. All involved studies used at least one measurement of balance, gait, or postural control as the outcome.

**Results:** A total of 145 studies were found, of which 10 (*n* = 246) met the inclusion criteria and included in our studies. The present meta-analysis showed that active tDCS have beneficial effects on timed up and go test (TUGT) [mean difference (MD): 0.35; 95% confidence interval (CI): 0.11 to 0.58] and Functional Ambulation Category (FAC) (MD: −2.54; 95% CI: −3.93 to −1.15) in stroke patients. However, the results were not significant on the berg balance scale (BBS) (MD: −0.20; 95% CI: −1.44 to 1.04), lower extremity subscale of Fugl-Meyer Assessment (FMA-LE) (MD: −0.43; 95% CI: −1.70 to 0.84), 10-m walk test (10 MWT) (MD: −0.93; 95% CI: −2.68 to 0.82) and 6-min walking test (6 MWT) (MD: −2.55; 95% CI: −18.34 to 13.23).

**Conclusions:** In conclusion, we revealed that tDCS might be an effective option for restoring walking independence and functional ambulation for stroke patients in our systematic review and meta-analysis.

**Systematic Review Registration:** CRD42020207565.

## Introduction

Stroke is a severe central nervous system disease caused by cerebral ischemia or hemorrhage. Stroke often causes multiple health problems, such as decreased muscle strength, impaired proprioceptive capabilities, and impaired cognitive function (1, 2). These sensorimotor deficits will further affect the balance and postural control of stroke patients, which is the key to keeping the body upright and stable in different environments and conditions (3). Nearly 80% of stroke patients cannot successfully maintain balance and execute postural control (4). Another study has shown that 38% of stroke patients are still non-ambulatory at 6 months after onset (5). These patients show weight-bearing asymmetry toward the non-paretic leg, increased postural swing, abnormal joint movement, and cannot adjust their posture (6, 7). Besides, chronic stage stroke can also limit walking ability recovery and increase the risk of falling (8). Therefore, the recovery of balance and postural control is essential for patients to improve their activities of daily living, quality of life and prevent fall events after stroke (9).

Standard rehabilitation protocols focus on improving the patient's walking ability and using compensation strategies like a wheelchair or crutches to maintain balance. In the past several years, therapies based on motor learning and modulating neuroplasticity have greatly been developed. Treatments that used biofeedback training and repetitive task-specific training seem to be effective for patient activities (10). Still, both training types do not demonstrate superior results on clinical tests of balance capacity because the improvement of neural plasticity needs enough training intensity (11, 12). Such limitations in treatment have prompted researchers to find new options. Transcranial direct current stimulation (tDCS) is a promising, innovative, non-invasive method of neurostimulation in which a weak direct current (about 1–2 mA) is applied on the scalp with electrodes. The effect of tDCS is polarity specific, in which anodal stimulation causes an increase in the excitability of the motor cortex, and a cathode causes it to decrease (13).

The safety and effectiveness of tDCS technology have been proven in treating cognitive impairment, depression, and pain (14). It has also been confirmed that tDCS can improve motor learning and precision in healthy people (15) and improve upper limb function and fine motor control in stroke patients (16, 17). Similarly, Madhavan et al. (18) demonstrated that stroke patients' ankle movement had been significantly modulated after excitatory stimulation of the ipsilesional motor cortex. Sohn et al. (19) reported that excitatory tDCS could enhance postural stability in patients with sub-acute stroke, but a sham stimulation cannot. Although many current studies have shown positive results on the effect of tDCS. However, some studies showed the opposite results (20). These inconsistencies might be related to the lower sample size, the difference in evaluation methods, and the patient's pre-intervention status. In this study, we aimed to make a systematic review and meta-analysis to evaluate the effect of tDCS on balance following stroke patients.

## Methods

### Search Strategy

We conducted this meta-analysis to explore the effect of tDCS on balance in stroke patients following the Preferred Reporting Items for Systematic reviews and Meta-Analysis (PRISMA) guideline (21). Online databases (PubMed, Web of Science, EMBASE, Cochrane Central Register of Controlled Trials, and Google Scholar) were searched for articles until August 2020. We performed the literature search for studies that used tDCS to improve balance, gait, and postural control in stroke patients. The key search terms were: [“transcranial direct current stimulation” (MeSH term)] AND [“stroke” (MeSH term)] AND [“gait” (MeSH term) OR “Postural Balance” (MeSH term)]. The keyword “OR” was used for the combination of the MeSH term and Entry terms.

### Inclusion Criteria and Exclusion Criteria

Our study followed these inclusion criteria: (1) randomized controlled trials (RCTs), crossover RCTs, and high-quality comparative studies focused on the tDCS effects in stroke patients' balance; (2) included at least one measurement of balance, gait, or postural control as the outcome; and (3) with sham stimulation as control.

The articles were excluded if they (1) were meta-analyses, reviews, study protocols, meeting abstracts, or case studies; (2) were animal experiments; and (3) had no sufficient data to calculate the effect size.

### Data Collection

We extracted data from the studies as follows: authors and publication year, the type of study design, numbers of objects, the site of stimulation, sessions, characteristics of interventions and controls, and measured outcomes. If the outcome data were reported at multiple time points, we used those obtained immediately after the intervention.

The following outcome measures were chosen for our meta-analysis: (1) lower extremity subscale of Fugl-Meyer Assessment (FMA-LE) that tests the recovery of lower extremity motor impairment, (2) Berg Balance Scale (BBS) that examines the balance function, (3) Functional Ambulatory Category (FAC) that tests walking independence and functional ambulation, (4) timed up and go test (TUGT) that tests functional mobility, (5) 10-m walk test (10 MWT) that tests the effect of tDCS on walking speed, and (6) 6-min walking test (6 MWT) that tests the walking endurance.

### Quality Assessment

We assessed the methodological quality of the included articles that follows the Cochrane Risk of Bias Tool (22). Two authors independently rated studies based on six domains: (1) sequence generation, (2) allocation concealment, (3) blinding of the participants, (4) blinding of the assessors, (5) method of addressing incomplete outcome data, and (6) selective reporting. The methodological quality assessment results have three levels in each domain: low risk, high risk, and unclear. We resolved any discrepancies during interpretation by discussion and mutual agreement.

### Meta-Analysis

This meta-analysis used means and standard deviations of the difference between pre-intervention and post-intervention. When the means and standard deviations had not been directly provided, we used *t*-values, F-values, or *p*-values to calculate effect sizes. We conducted the pooled effect size calculation by using mean differences (MD) according to the outcome measures of studies. Heterogeneity between studies was assessed based on I2 statistics. We used a random-effects model when the threshold for heterogeneity was above 50%. Then, a sensitivity analysis was performed to control the heterogeneity by excluding one study at a time. When the heterogeneity threshold is below 50%, we used a fixed-effects model for the calculation. All statistical analyses were conducted using Review Manager 5.4.

## Results

### Search Results

**Figure 1:**
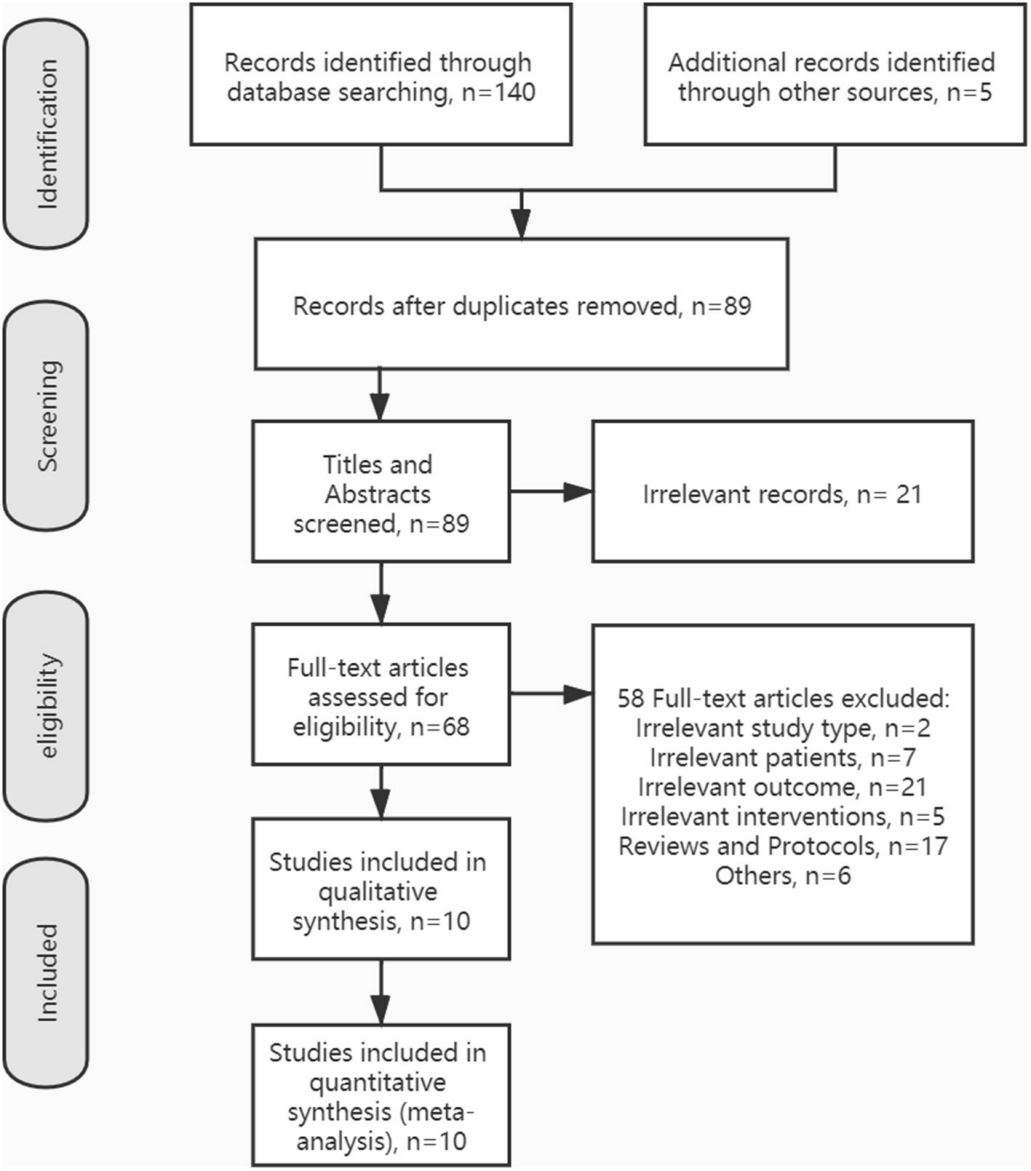
Preferred reporting items for systematic reviews and meta-analysis (PRISMA) flow diagram showing the literature search, inclusion, and exclusion process.

The process of inclusion and exclusion is summarized in Figure 1. Finally, 10 articles were included for our systematic reviews and meta-analysis. Table 1 shows the characteristics of the included studies and their primary outcome measures. Seven RCTs, (23–25, 27–29, 32) two crossovers, (26, 31) and one active-control article, (30) with a total of 246 stroke patients, were included in this study. Among all the included literature, there were five articles that reported on TUGT, (23, 26–28, 31) five articles reported on FAC, (24, 28–30, 32) four articles reported on 10 MWT, (24, 26, 28, 32) four articles reported on 6MWT, (24, 25, 27, 32) four articles reported on BBS, (24, 27–29) four articles reported FMA-LE, (24, 26, 27, 29) three articles reported CME, (24, 27, 29) two articles reported on walking speed, (27, 30) two articles reported on POMA, (23, 26) and one article reported on MVC and FTSST (31). For those outcome measures reported in more than three articles, we calculated the pooled effect size.

**Table 1:** Main characteristics and outcomes of the reviewed articles.

**Authors**	**Study design**	**Numbers of objects (M/F)**	**Area of application**		**Number of sessions**	**tDCS parameters**	**Additional activity**	**Outcome measures**
			**Active electrode**	**Reference electrode**				
Tahtis et al. (23)	RCT	11/3	Ipsilesional leg motor area	Contralesional leg motor area	1	25 cm^2^ 2 mA 15 min		POMA; TUGT
Seo et al. (24)	RCT	16/5	Ipsilesional leg motor area	Forehead above the contralateral orbit	10	35 cm^2^ 2 mA 20 min	RAGT	FAC; 10 MWT; CME (MEP); 6 MWT; BBS; FMA-LE
Picelli et al. (25)	RCT	15/5	Ipsilesional M1	Contralateral orbit	10	35 cm^2^ 2.5 mA 20 min	RAGT	6 MWT; FAC
Manji et al. (26)	Crossover	21/9	SMA	Inion	NR	25 cm^2^ 1 mA 20 min	BWSTT	10 MWT; TUGT; FMA-LE; POMA
Madhavan et al. (27)	RCT	30/10	Ipsilesional leg motor area	Contralesional supraorbital area	12	12.5 cm^2^ 1 mA 15 min	AMT;HISTT	Walking speed (10 MWT); CME (MEP);6 MWT; BBS; FMA-LE; TUGT
Danzl et al. (28)	RCT	4/4	Ipsilesional leg motor area	Supraorbital area	12	25 cm^2^ 2 mA 20 min	LT-RGO	10 MWT; TUGT; FAC; BBS
Chang et al. (29)	RCT	15/9	Determined using TMS	Forehead above the contralateral supraorbital area	10	7.07 cm^2^ 2 mA 10 min	Conventional physical therapy	CME (MEP); FMA-LE; BBS; FAC
Leon et al. (30)	Active control	35/15	Ipsilesional leg and hand motor area	Contralateral supraorbital area	20	35 cm^2^ 2 mA 20 min	RAGT	Walking speed (10 MWT); FAC
Klomjai et al. (31)	Crossover	14/5	Ipsilesional M1	Contralesional M1	1	35 cm^2^ 2 mA 20 min	Conventional physical therapy	MVC (knee extensor); TUGT; FTSST
Geroin et al. (32)	RCT	14/6	Ipsilesional leg motor area	Contralateral orbit of the eye	10	35 cm^2^ 1.5 mA 7 min	RAGT	6 MWT; 10 MWT; FAC

*AMT, ankle motor tracking; BWSTT, body weight-supported treadmill training; HISTT, high-intensity speed-based treadmill training; LT-RGO, locomotor training with a robotic gait orthosis; M1, primary motor cortex; MEP, motor-evoked potential; NR, not reported; RAGT, robot-assisted gait training; SMA, supplementary motor area; TMS, transcranial magnetic stimulation*.

### Quality Assessments

**Figure 2:**
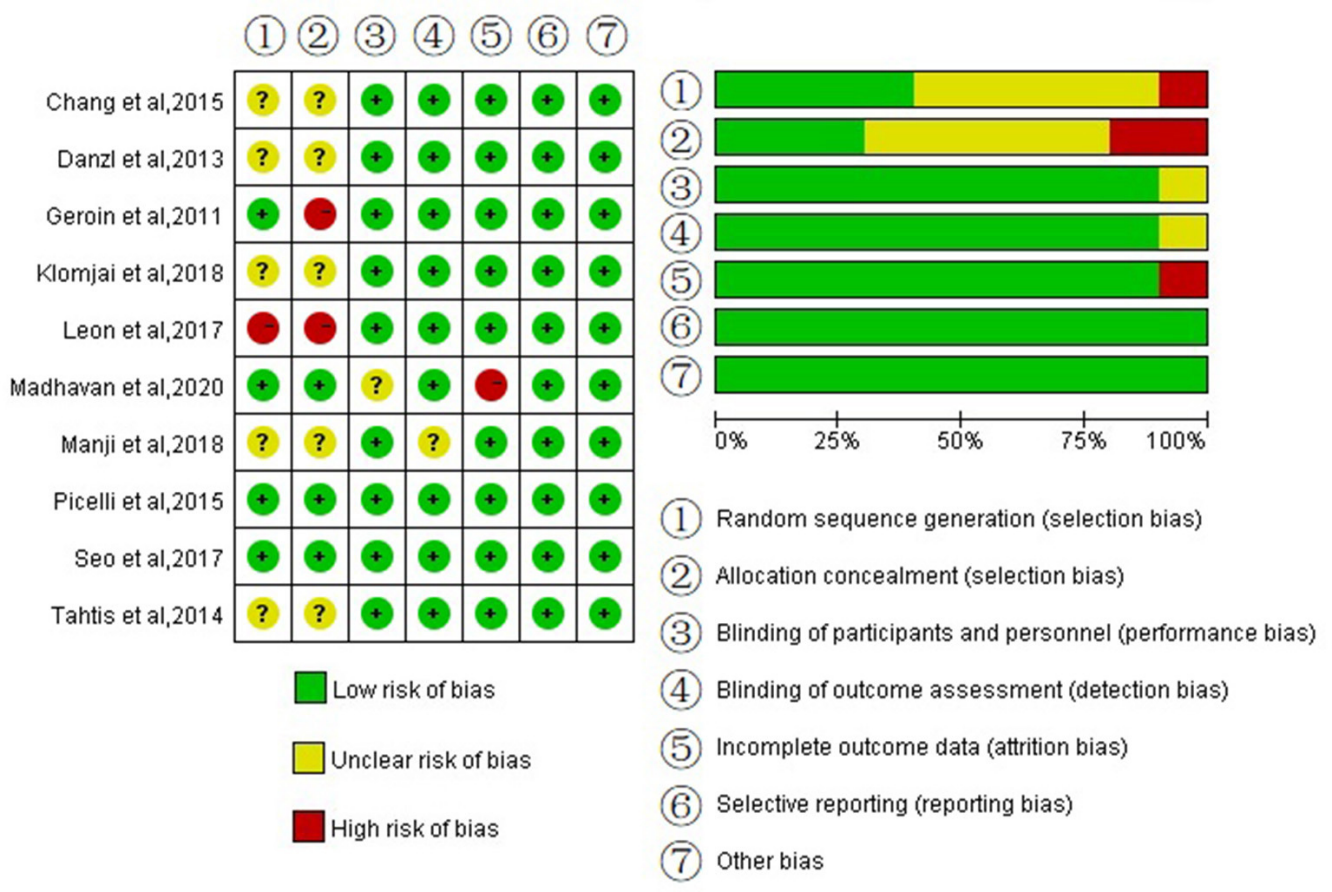
Risk of bias graph, according to the cochrane criteria.

Figure 2 shows the methodological qualities of the trials with Cochrane recommendation. Among all the articles, one study had participants sequentially assigned to one of two groups, (30) and four studies clearly described the method of random allocation [two according to software-generated randomization scheme, (25, 32) one used the minimization method, (27) one used random table (24)], and the remaining studies only mentioned randomization without describing the specific implementation plan. Three studies described certain schemes of allocation concealment (24, 25, 27). Two studies had not sufficiently reported the blinding of participants and the blinding of outcome assessment (26, 27). One study had missing outcome data (27). None of the studies showed selective reporting results and other biases. Overall, the included studies were at a relatively low risk of bias.

### Meta-Analysis Results

#### Lower Extremity Subscale of Fugl-Meyer Assessment

**Figure 3:**
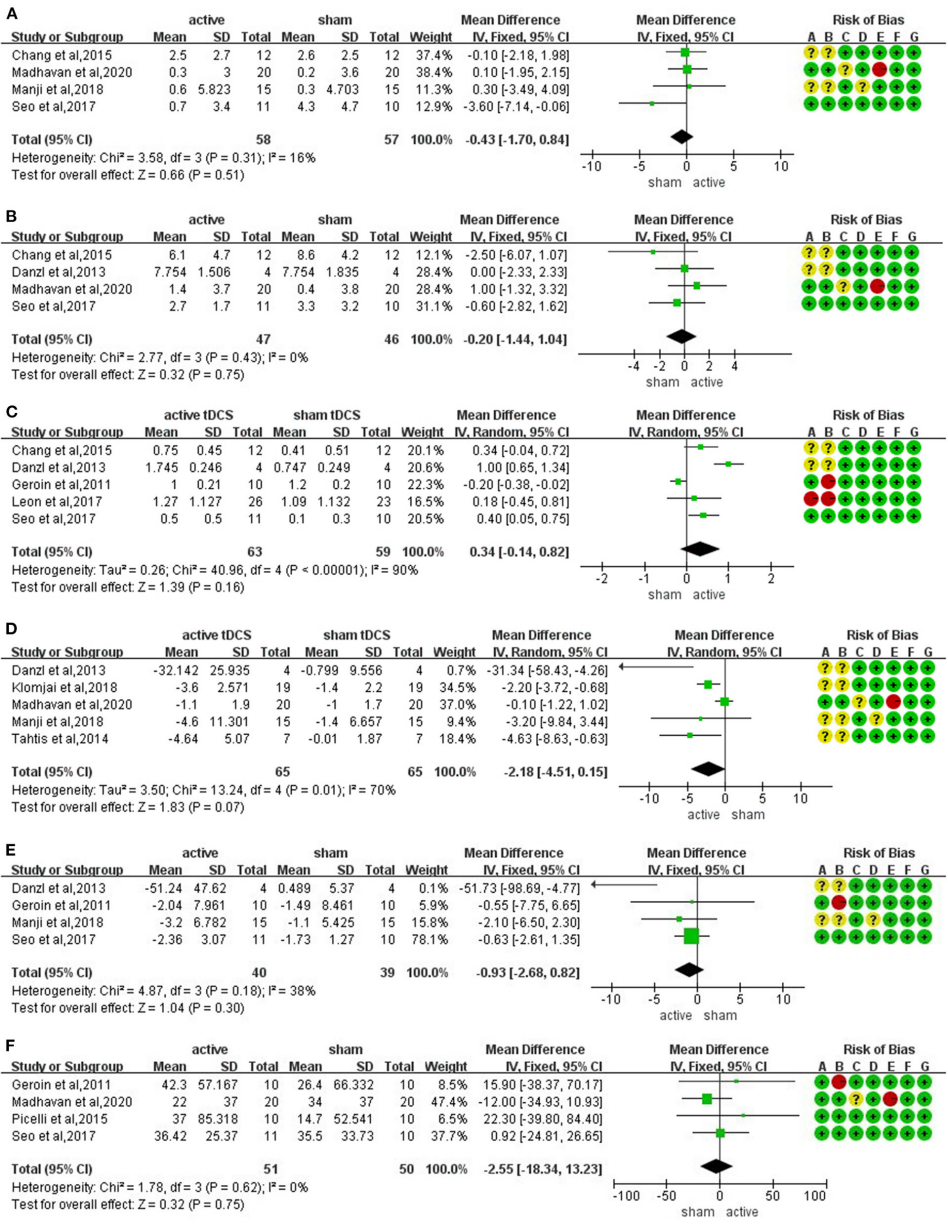
Forrest plots displaying the mean differences (MD) between active and sham groups. **(A)** Lower extremity subscale of Fugl-Meyer Assessment (FMA-LE). **(B)** berg balance scale (BBS). **(C)** functional ambulation category (FAC). **(D)** timed up and go test (TUGT). **(E)** 10-m walk test (10 MWT). **(F)** 6-min walking test (6 MWT).

The meta-analysis of four trials and 115 participants showed no significant difference on FMA-LE in active tDCS compared with sham tDCS (MD: −0.43; 95% CI: −1.70 to 0.84; *p* = 0.51; I2 = 16%; inverse variance method with fixed-effects model) (Figure 3A).

#### Berg Balance Scale

The meta-analysis of four trials and 93 participants showed no significant difference on BBS in active tDCS compared with sham tDCS (MD: −0.20; 95% CI: −1.44 to 1.04; *p* = 0.75; I2 = 0%; inverse variance method with fixed-effects model) (Figure 3B).

#### Functional Ambulation Category

**Figure 4:**
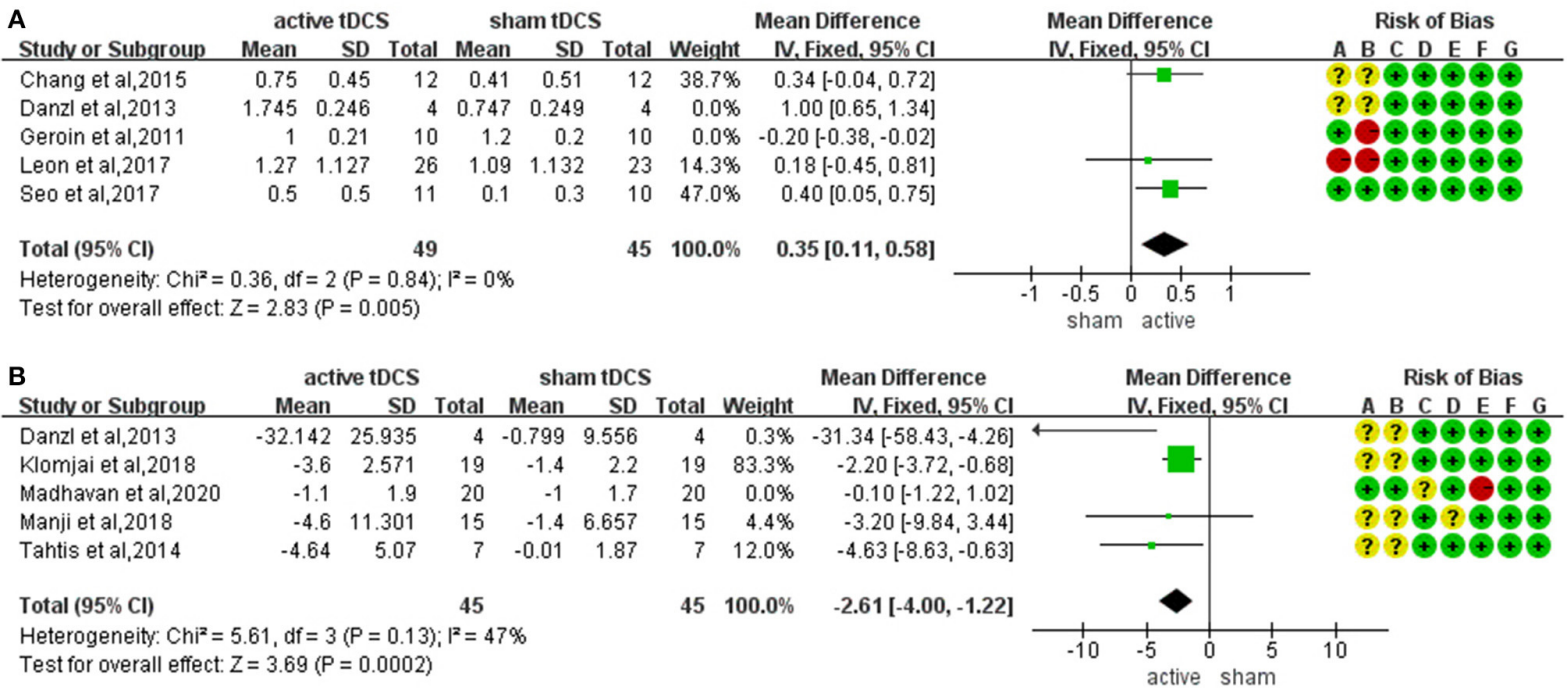
Sensitivity analysis for the FAC **(A)** and TUGT **(B)**.

The meta-analysis of five trials and 122 participants showed no significant difference on FAC in active tDCS compared with sham tDCS (MD: 0.34; 95% CI: −0.14 to 0.82; *p* = 0.16; I2 = 90%; inverse variance method with random-effects model) (Figure 3C). The studies presented a high heterogeneity (I2 ≥ 50%). A sensitivity analysis was performed by excluding one study at a time. Two studies were excluded achieving I2 = 0%. The result showed a significant improvement on FAC scores in active tDCS compared with sham tDCS (MD: 0.35; 95% CI: 0.11 to 0.58; *p* = 0.005; I2 = 0%; inverse variance method with fixed-effects model) (Figure 4A).

#### Timed Up and Go Test

The meta-analysis of five trials and 130 participants showed no significant difference on TUGT in active tDCS compared with sham tDCS (MD: −2.18; 95% CI: −4.51 to 0.15; *p* = 0.07; I2 = 70%; inverse variance method with random-effects model) (Figure 3D). The studies presented a high heterogeneity (I2 ≥ 50%). A sensitivity analysis was performed by excluding one study at a time. One study was excluded achieving I2 = 47%. The result showed a significant reduction in timing when performing TUGT in active tDCS compared with sham tDCS (MD: −2.61; 95% CI: −4.00 to −1.22; *p* = 0.0002; I2 = 47%; inverse variance method with fixed-effects model) (Figure 4B).

#### 10-m Walking Test

The meta-analysis of four trials and 79 participants showed no significant difference on 10 MWT in active tDCS compared with sham tDCS (MD: −0.93; 95% CI: −2.68 to 0.82; *p* = 0.30; I2 = 38%; inverse variance method with fixed-effects model) (Figure 3E).

#### 6-min Walking Test

The meta-analysis of four trials and 101 participants showed no significant difference on 6 MWT in active tDCS compared with sham tDCS (MD: −2.55; 95% CI: −18.34 to 13.23; *p* = 0.75; I2 = 0%; inverse variance method with fixed-effects model) (Figure 3F).

## Discussion

The present study included 10 trials to assess the effects of tDCS on balance capacity in stroke patients. Meta-analysis results showed an overall improvement in FAC and TUGT after implementing active tDCS, but no significant effects were found on FMA-LE, BBS, 10 MWT, and 6 MWT. The Functional Ambulation Category is used to classify locomotion ability depending on how much assistance the subject requires from others when walking (33, 34). Higher scores indicate better walking independence and functional ambulation. The TUGT tests functional mobility and correlates well with gait performance, lower limb weight-bearing, and walking endurance in stroke (35, 36). Although our studies do not directly show a significant effect of tDCS on balance, active tDCS still has some positive effects on walking independence, gait, and ambulation when compared with sham tDCS.

A decrease in cortical excitability often accompanies the occurrence of stroke. The decrease in cortical excitability is usually associated with a lesion of the affected brain or excessive inhibition from the contralateral hemisphere, or both. Previous studies have shown that the reorganization of motor circuits in the cerebral cortex after stroke was more crucial than the neural repair process (37). Therefore, regulating cortical excitability, activating latent neural pathways, or enhancing the efficiency of neural connection to accelerate brain function remodeling is the key to promote the recovery of motor function in stroke patients. So far, several studies have studied the effect of tDCS on the excitability of the motor cortex of lower limbs. Nitsche et al. discovered that tDCS could improve humans' cortical plasticity and motor function in stroke patients (13). Similarly, serval related studies also showed that tDCS applied to the lower limb representative area of the motor cortex could improve ankle fine motor control, (18) motor adaptability, (38) quadriceps muscle strength, (39) and increase the evoked potential amplitude of anterior tibial muscle (40).

Different from the unilateral cortical dominance of the upper limb, the bilateral hemispheres control the lower limb (41). Therefore, the hyperexcitability of the un-lesioned hemisphere relative to the lesioned hinders the lower limb's functional recovery after stroke. Relevant studies have also indicated that gait and balance performance in stroke populations depends on intact interhemispheric connections, (42) and asymmetry between cerebral hemispheres could be used as a prognostic indicator of motor recovery after stroke (43). As Enzinger et al. showed in their study, walking endurance in patients with chronic stroke could be improved by facilitating the bilateral hemispheric motor cortex, cingulate gyrus motor area, and caudate nucleus (44). This suggested that bilateral stimulation might be better than unilateral stimulation in improving balance and gait. Prior studies have found that bilateral tDCS stimulation was better than unilateral tDCS in regulating motor excitability (45), and bilateral tDCS could better reduce stroke patients' fall risk than unilateral tDCS (46). However, further studies are still needed to explore the detailed mechanism of bilateral tDCS in the rehabilitation of balance following stroke.

The underlying mechanism of tDCS in regulating stroke patients' balance function is related to cortical excitability. Weak direct current can lead to subthreshold regulation of membrane potential and regulates the resting membrane potentials toward depolarization or hyperpolarization (14), which affects cortical excitability. Besides, the interhemispheric imbalance after stroke will hinder the recovery of patients' functions. The use of tDCS (especially bilateral tDCS) can restore the two hemispheres' symmetry and improve the stroke population's gait and balance (42). The cerebellum plays an essential role in coordinating limb movements and maintaining body balance. Naro et al. found that cerebellar stimulation could activate the Purkinje cells, intensify its inhibition to cerebellar nuclei, and weaken the cerebral cortex's abnormal excitement (47). When the cerebellum is more involved in the movement's planning and coordination process, it improves the patient's balance function. There is an off-line therapeutic effect of tDCS, which is related to the regulation of synaptic plasticity. The main presentation is long-term potentiation (LTP) and long-term inhibition (LTP) mediated by the N-methyl-D-aspartic acid receptor (NMDA) system (48). Decreased regional cerebral blood flow (rCBF) after stroke is related to postural instability and increases the risk of falls (49). Studies have shown that when the dorsolateral prefrontal cortex (DLPFC) is stimulated by anodal tDCS, rCBF under the electrodes can be increased (50). In contrast, the rCBF significantly decreases under cathode, suggesting that tDCS can improve stroke patients' postural stability and balance function by increasing rCBF.

The current results are also consistent with previous meta-analyses (51), which assessed the effects of tDCS to improve the ambulation ability in stroke patients. The authors included 14 articles with 266 patients in their study, which showed that the FAC and TUGT improved significantly following active tDCS, but the effects on walking speed and walking endurance (6 MWT) were not significant in their study. Similarly, Li et al. revealed that the effects of tDCS on mobility and muscle strength after stroke were statistically significant, despite the results were not significant on balance function (SMD: 0.44; 95% CI: −0.06 to 0.94). (52). Accounting for the non-significant effect size on balance function may be because the maintenance of balance requires multiple factors (visual acuity, proprioception, inner ear function, foot position on the ground, etc.) that are not clear whether they are influenced by tDCS or not (19). In this case, tDCS alone is challenging to improve stroke patients' balance ability, and further studies are needed to evaluate all the factors affecting balance performance. Balance ability measured by BBS was considered the strongest predictor of 10-m and 6-min walking in stroke patients (53). Since tDCS cannot improve balance function, walking speed and walking endurance will also be limited. Besides, this study does not find that tDCS has more advantages in improving lower extremity motor impairment (FMA-LE) in stroke patients, which may be owed to motor impairment after stroke needs a long time to recover. However, this meta-analysis only includes the data immediately after the intervention. At present, the application and research of tDCS after stroke are gradually increasing. The application of tDCS requires the definition of multiple parameters, including the intensity of the stimulation current, duration, number of stimulations, position, and size of the electrodes. Through the combination of different parameters, the effects of tDCS are not the same. There are no unified standards for selecting stimulation parameters in clinical use, and careful considerations such as specific lesion locations, duration of onset, and safety are required. From the perspective of rehabilitation, tDCS, as an adjuvant treatment method, can be combined with functional training such as robotic-assisted gait training (RAGT), (24) constraint-induced movement therapy (CIMT), (54) and virtual reality (55–58), which combines neuromodulation and behavioral intervention to improve the patient's function. In sum, for patients with balance dysfunction after stroke, we should choose appropriate stimulation parameters and combine tDCS stimulation with traditional rehabilitation programs. These may help restore the balance function of stroke patients and make them back to society.

Several limitations should be noted when interpreting the results. (1) The long-term effect of tDCS on balance in stroke patients was not considered. (2) Since some trials did not provide the data directly, or the author did not reply to our data request, part of the data was extracted from the figures. The results may be biased. (3) Only the articles published in English were included. (4) The subgroup analyses were hard to implement because the number of included studies is small. (5) It was not considered the publication bias in the case of a small amount of included literature. In this META analysis, we explored the effects of tDCS on balance and gait in stroke patients based on multiple measurement indicators. In summary, the main conclusion drawn from our meta-analysis is that active tDCS have beneficial effects on FAC and TUGT in stroke patients (i.e., to improve walking independence and functional ambulation). Still, the improvement is not significant in the recovery of lower extremity motor impairment, walking endurance, gait speed, and overall balance function. Future research should focus on reproducing the results in a larger sample size and a long-time follow-up to understand the role of cognitive function in maintaining gait and balance in stroke patients and how tDCS can improve the balance function by affecting cognition. Furthermore, in future studies, we need to consider a variety of neurophysiological results, the effects of various factors on balance, and the use of imaging techniques.

## Data Availability Statement

The original contributions presented in the study are included in the article/supplementary material, further inquiries can be directed to the corresponding author/s.

## Author Contributions

KD: conceptualization, methodology, formal analysis, and writing-original draft. SM: quality assessment and project administration. ZG: quality assessment and formal analysis. RZ, PX, and EY: investigation and data curation. TL: validation, supervision, writing-reviewing, and editing. All authors contributed to the article and approved the submitted version.

## Conflict of Interest

The authors declare that the research was conducted in the absence of any commercial or financial relationships that could be construed as a potential conflict of interest.

## Abbreviations

10 MWT: 10-m walk test
6 MWT: 6-min walking test
BBS: berg balance scale
CME: corticomotor excitability
FAC: functional ambulation category
FMA-LE: lower extremity of Fugl-Meyer assessment
FTSST: five-times-sit-to-stand
MVC: maximum voluntary contraction
POMA: performance-oriented mobility assessment
RCT: randomized controlled trial
TUGT: timed up and go test.

## References

[1] Benjamin EJ Blaha MJ Chiuve SE Cushman M Das SR Deo R. Heart disease and stroke statistics-2017 update: a report from the American Heart Association. Circulation. (2017) 135:e146–603. 10.1161/CIR.0000000000000491 28122885 PMC5408160

[2] Smania N Picelli A Gandolfi M Fiaschi A Tinazzi M. Rehabilitation of sensorimotor integration deficits in balance impairment of patients with stroke hemiparesis: a before/after pilot study. Neurol Sci. (2008) 29:313–9. 10.1007/s10072-008-0988-0 18941933

[3] Balaban B Tok F. Gait disturbances in patients with stroke. PM R. (2014) 6:635–42. 10.1016/j.pmrj.2013.12.017 24451335

[4] Tyson SF Hanley M Chillala J Selley A Tallis RC. Balance disability after stroke. Phys Ther. (2006) 86:30–8. 10.1093/ptj/86.1.30 16386060

[5] Kollen B Kwakkel G Lindeman E. Longitudinal robustness of variables predicting independent gait following severe middle cerebral artery stroke: a prospective cohort study. Clin Rehabil. (2006) 20:262–8. 10.1191/0269215506cr910oa 16634346

[6] Hugues A Di Marco J Janiaud P Xue Y Pires J Khademi H. Efficiency of physical therapy on postural imbalance after stroke: study protocol for a systematic review and meta-analysis. BMJ Open. (2017) 7:e013348. 10.1136/bmjopen-2016-013348 28137928 PMC5293873

[7] Kerrigan DC Gronley J Perry J. Stiff-legged gait in spastic paresis. A study of quadriceps and hamstrings muscle activity. Am J Phys Med Rehabil. (1991) 70:294–300. 10.1097/00002060-199112000-00003 1741998

[8] Batchelor FA Williams SB Wijeratne T Said CM Petty S. Balance and gait impairment in transient ischemic attack and minor stroke. J Stroke Cerebrovasc Dis. (2015) 24:2291–7. 10.1016/j.jstrokecerebrovasdis.2015.06.014 26227322

[9] Geurts ACH de Haart M van Nes IJW Duysens J. A review of standing balance recovery from stroke. Gait Posture. (2005) 22:267–81. 10.1016/j.gaitpost.2004.10.002 16214666

[10] Lee CH Kim Y Lee BH. Augmented reality-based postural control training improves gait function in patients with stroke: Randomized controlled trial. Hong Kong Physiother J. (2014) 32:51–7. 10.1016/j.hkpj.2014.04.002

[11] Lee SH Byun SD Kim CH Go JY Nam HU Huh JS. Feasibility and effects of newly developed balance control trainer for mobility and balance in chronic stroke patients: a randomized controlled trial. Ann Rehabil Med. (2012) 36:521–9. 10.5535/arm.2012.36.4.521 22977778 PMC3438419

[12] de Rooij IJM van de Port IGL Meijer J-WG. Effect of virtual reality training on balance and gait ability in patients with stroke: systematic review and meta-analysis. Phys Ther. (2016) 96:1905–18. 10.2522/ptj.20160054 27174255

[13] Nitsche MA Paulus W. Sustained excitability elevations induced by transcranial DC motor cortex stimulation in humans. Neurology. (2001) 57:1899–901. 10.1212/WNL.57.10.1899 11723286

[14] Lefaucheur J-P Antal A Ayache SS Benninger DH Brunelin J Cogiamanian F. Evidence-based guidelines on the therapeutic use of transcranial direct current stimulation (tDCS). Clin Neurophysiol. (2017) 128:56–92. 10.1016/j.clinph.2016.10.087 27866120

[15] Nitsche MA Schauenburg A Lang N Liebetanz D Exner C Paulus W. Facilitation of implicit motor learning by weak transcranial direct current stimulation of the primary motor cortex in the human. J Cogn Neurosci. (2003) 15:619–26. 10.1162/089892903321662994 12803972

[16] Kang N Summers JJ Cauraugh JH. Transcranial direct current stimulation facilitates motor learning post-stroke: a systematic review and meta-analysis. J Neurol Neurosurg Psychiatry. (2016) 87:345–55. 10.1136/jnnp-2015-311242 26319437

[17] Tedesco Triccas L Burridge JH Hughes AM Pickering RM Desikan M Rothwell JC. Multiple sessions of transcranial direct current stimulation and upper extremity rehabilitation in stroke: a review and meta-analysis. Clin Neurophysiol. (2016) 127:946–55. 10.1016/j.clinph.2015.04.067 25998205

[18] Madhavan S Weber KA 2nd Stinear JW. Non-invasive brain stimulation enhances fine motor control of the hemiparetic ankle: implications for rehabilitation. Exp Brain Res. (2011) 209:9–17. 10.1007/s00221-010-2511-0 21170708

[19] Sohn MK Jee SJ Kim YW. Effect of transcranial direct current stimulation on postural stability and lower extremity strength in hemiplegic stroke patients. Ann Rehabil Med. (2013) 37:759–65. 10.5535/arm.2013.37.6.759 24466510 PMC3895515

[20] Steiner KM Enders A Thier W Batsikadze G Ludolph N Ilg W. Cerebellar tDCS does not improve learning in a complex whole body dynamic balance task in young healthy subjects. PLoS ONE. (2016) 11:e0163598. 10.1371/journal.pone.0163598 27669151 PMC5036893

[21] Moher D Liberati A Tetzlaff J Altman DG. Preferred reporting items for systematic reviews and meta-analyses: the PRISMA statement. BMJ. (2009) 339:b2535. 10.1136/bmj.b2535 19622551 PMC2714657

[22] Higgins JPT Thomas J Chandler J Cumpston M Li T Page MJ editors. Cochrane Handbook for Systematic Reviews of Interventions. 2nd ed. Chichester: John Wiley & Sons (2019).

[23] Tahtis V Kaski D Seemungal BM. The effect of single session bi-cephalic transcranial direct current stimulation on gait performance in sub-acute stroke: a pilot study. Restor Neurol Neurosci. (2014) 32:527–32. 10.3233/RNN-140393 24906374

[24] Seo HG Lee WH Lee SH Yi Y Kim KD Oh B-M. Robotic-assisted gait training combined with transcranial direct current stimulation in chronic stroke patients: A pilot double-blind, randomized controlled trial. Restor Neurol Neurosci. (2017) 35:527–36. 10.3233/RNN-170745 28800341

[25] Picelli A Chemello E Castellazzi P Roncari L Waldner A Saltuari L. Combined effects of transcranial direct current stimulation (tDCS) and transcutaneous spinal direct current stimulation (tsDCS) on robot-assisted gait training in patients with chronic stroke: a pilot, double blind, randomized controlled trial. Restor Neurol Neurosci. (2015) 33:357–68. 10.3233/RNN-140474 26410579

[26] Manji A Amimoto K Matsuda T Wada Y Inaba A Ko S. Effects of transcranial direct current stimulation over the supplementary motor area body weight-supported treadmill gait training in hemiparetic patients after stroke. Neurosci Lett. (2018) 662:302–5. 10.1016/j.neulet.2017.10.049 29107706

[27] Madhavan S Cleland BT Sivaramakrishnan A Freels S Lim H Testai FD. Cortical priming strategies for gait training after stroke: a controlled, stratified trial. J Neuroeng Rehabil. (2020) 17:111. 10.1186/s12984-020-00744-9 32799922 PMC7429759

[28] Danzl MM Chelette KC Lee K Lykins D Sawaki L. Brain stimulation paired with novel locomotor training with robotic gait orthosis in chronic stroke: a feasibility study. NeuroRehabilitation. (2013) 33:67–76. 10.3233/NRE-130929 23949035 PMC4349529

[29] Chang MC Kim DY Park DH. Enhancement of cortical excitability and lower limb motor function in patients with stroke by transcranial direct current stimulation. Brain Stimul. (2015) 8:561–6. 10.1016/j.brs.2015.01.411 25736569

[30] Leon D Cortes M Elder J Kumru H Laxe S Edwards DJ. tDCS does not enhance the effects of robot-assisted gait training in patients with subacute stroke. Restor Neurol Neurosci. (2017) 35:377–84. 10.3233/RNN-170734 28697574

[31] Klomjai W Aneksan B Pheungphrarattanatrai A Chantanachai T Choowong N Bunleukhet S. Effect of single-session dual-tDCS before physical therapy on lower-limb performance in sub-acute stroke patients: a randomized sham-controlled crossover study. Ann Phys Rehabil Med. (2018) 61:286–91. 10.1016/j.rehab.2018.04.005 29763676

[32] Geroin C Picelli A Munari D Waldner A Tomelleri C Smania N. Combined transcranial direct current stimulation and robot-assisted gait training in patients with chronic stroke: a preliminary comparison. Clin Rehabil. (2011) 25:537–48. 10.1177/0269215510389497 21402651

[33] Holden MK Gill KM Magliozzi MR. Gait assessment for neurologically impaired patients. Standards for outcome assessment. Phys Ther. (1986) 66:1530–9. 10.1093/ptj/66.10.1530 3763704

[34] Mehrholz J Wagner K Rutte K Meissner D Pohl M. Predictive validity and responsiveness of the functional ambulation category in hemiparetic patients after stroke. Arch Phys Med Rehabil. (2007) 88:1314–9. 10.1016/j.apmr.2007.06.764 17908575

[35] Podsiadlo D Richardson S. The timed “Up & Go”: a test of basic functional mobility for frail elderly persons. J Am Geriatr Soc. (1991) 39:142–8. 10.1111/j.1532-5415.1991.tb01616.x 1991946

[36] Ng SS Hui-Chan CW. The timed up & go test: its reliability and association with lower-limb impairments and locomotor capacities in people with chronic stroke. Arch Phys Med Rehabil. (2005) 86:1641–7. 10.1016/j.apmr.2005.01.011 16084820

[37] Ward N. Assessment of cortical reorganisation for hand function after stroke. J Physiol. (2011) 589:5625–32. 10.1113/jphysiol.2011.220939 22063630 PMC3249038

[38] Kaski D Quadir S Patel M Yousif N Bronstein AM. Enhanced locomotor adaptation aftereffect in the “broken escalator” phenomenon using anodal tDCS. J Neurophysiol. (2012) 107:2493–505. 10.1152/jn.00223.2011 22323638 PMC3362242

[39] Tanaka S Takeda K Otaka Y Kita K Osu R Honda M. Single session of transcranial direct current stimulation transiently increases knee extensor force in patients with hemiparetic stroke. Neurorehabil Neural Repair. (2011) 25:565–9. 10.1177/1545968311402091 21436391

[40] Jeffery DT Norton JA Roy FD Gorassini MA. Effects of transcranial direct current stimulation on the excitability of the leg motor cortex. Exp Brain Res. (2007) 182:281–7. 10.1007/s00221-007-1093-y 17717651

[41] Luft AR Smith GV Forrester L Whitall J Macko RF Hauser T-K. Comparing brain activation associated with isolated upper and lower limb movement across corresponding joints. Hum Brain Mapp. (2002) 17:131–40. 10.1002/hbm.10058 12353246 PMC6872124

[42] Sullivan EV Adalsteinsson E Hedehus M Ju C Moseley M Lim KO. Equivalent disruption of regional white matter microstructure in ageing healthy men and women. Neuroreport. (2001) 12:99–104. 10.1097/00001756-200101220-00027 11201100

[43] Agius Anastasi A Falzon O Camilleri K Vella M Muscat R. Brain symmetry index in healthy and stroke patients for assessment and prognosis. Stroke Res Treat. (2017) 2017:8276136. 10.1155/2017/8276136 28251015 PMC5304313

[44] Enzinger C Dawes H Johansen-Berg H Wade D Bogdanovic M Collett J. Brain activity changes associated with treadmill training after stroke. Stroke. (2009) 40:2460–7. 10.1161/STROKEAHA.109.550053 19461018 PMC7610851

[45] Halakoo S Ehsani F Hosnian M Zoghi M Jaberzadeh S. The comparative effects of unilateral and bilateral transcranial direct current stimulation on motor learning and motor performance: A systematic review of literature and meta-analysis. J Clin Neurosci. (2020) 72:8–14. 10.1016/j.jocn.2019.12.022 31973922

[46] Andrade SM Ferreira JJ de A Rufino TS Medeiros G Brito JD da Silva MA. Effects of different montages of transcranial direct current stimulation on the risk of falls and lower limb function after stroke. Neurol Res. (2017) 39:1037–43. 10.1080/01616412.2017.1371473 28885111

[47] Naro A Bramanti A Leo A Manuli A Sciarrone F Russo M. Effects of cerebellar transcranial alternating current stimulation on motor cortex excitability and motor function. Brain Struct Funct. (2017) 222:2891–906. 10.1007/s00429-016-1355-1 28064346

[48] Cooke SF Bliss TVP. Plasticity in the human central nervous system. Brain. (2006) 129:1659–73. 10.1093/brain/awl082 16672292

[49] Fitzgibbon-Collins LK Noguchi M Heckman GA Hughson RL Robertson AD. Acute reduction in cerebral blood velocity on supine-to-stand transition increases postural instability in young adults. Am J Physiol Heart Circ Physiol. (2019) 317:H1342–53. 10.1152/ajpheart.00360.2019 31674810

[50] Stagg CJ Lin RL Mezue M Segerdahl A Kong Y Xie J. Widespread modulation of cerebral perfusion induced during and after transcranial direct current stimulation applied to the left dorsolateral prefrontal cortex. J Neurosci. (2013) 33:11425–31. 10.1523/JNEUROSCI.3887-12.2013 23843514 PMC3724554

[51] Tien H-H Liu W-Y Chen Y-L Wu Y-C Lien H-Y. Transcranial direct current stimulation for improving ambulation after stroke: a systematic review and meta-analysis. Int J Rehabil Res. (2020) 43:299–309. 10.1097/MRR.0000000000000427 32675686 PMC7643800

[52] Li Y Fan J Yang J He C Li S. Effects of transcranial direct current stimulation on walking ability after stroke: A systematic review and meta-analysis. Restor Neurol Neurosci. (2018) 36:59–71. 10.3233/RNN-170770 29439362

[53] Patterson SL Forrester LW Rodgers MM Ryan AS Ivey FM Sorkin JD. Determinants of walking function after stroke: differences by deficit severity. Arch Phys Med Rehabil. (2007) 88:115–9. 10.1016/j.apmr.2006.10.025 17207686

[54] Figlewski K Blicher JU Mortensen J Severinsen KE Nielsen JF Andersen H. Transcranial direct current stimulation potentiates improvements in functional ability in patients with chronic stroke receiving constraint-induced movement therapy. Stroke. (2017) 48:229–32. 10.1161/STROKEAHA.116.014988 27899754

[55] Massetti T Crocetta TB Silva TD da Trevizan IL Arab C Caromano FA. Application and outcomes of therapy combining transcranial direct current stimulation and virtual reality: a systematic review. Disabil Rehabil Assist Technol. (2017) 12:551–9. 10.1080/17483107.2016.1230152 27677678

[56] Kim YJ Ku J Cho S Kim HJ Cho YK Lim T. Facilitation of corticospinal excitability by virtual reality exercise following anodal transcranial direct current stimulation in healthy volunteers and subacute stroke subjects. J Neuroeng Rehabil. (2014) 11:1–12. 10.1186/1743-0003-11-124 25135003 PMC4148539

[57] Lee SJ Chun MH. Combination transcranial direct current stimulation and virtual reality therapy for upper extremity training in patients with subacute stroke. Arch Phys Med Rehabil. (2014) 95:431–8. 10.1016/j.apmr.2013.10.027 24239790

[58] Yao X Cui L Wang J Feng W Bao Y Xie Q. Effects of transcranial direct current stimulation with virtual reality on upper limb function in patients with ischemic stroke: a randomized controlled trial. J Neuroeng Rehabil. (2020) 17:1–8. 10.1186/s12984-020-00699-x 32539812 PMC7296643

